# Why small towns are shrinking: The spatial heterogeneity of small towns shrinkage and the impact of it from the perspective of rural-urban interaction in China

**DOI:** 10.1371/journal.pone.0293889

**Published:** 2023-11-02

**Authors:** Yong Han, Yating Deng, Ruixing Ni

**Affiliations:** School of Geographic Sciences, The Center of Targeted Poverty Alleviation and Rural Revitalization, Xinyang Normal University, Xinyang, China; Northeastern University (Shenyang China), CHINA

## Abstract

Small towns play a crucial role in bridging urban and rural territory systems. While numerous studies have identified the characteristics and causes of small town shrinkage (STS), there remains an unexplored perspective on the reasons for their shrinkage from the perspective of the rural-urban relationship. To address this research gap, we investigated the relationship between STS and rural-urban interaction (RUI) in China. We hypothesized that a negative relationship existed between the degree of STS and the intensity of RUI. Using geo-statistical methods, such as the multi-scale geographical weighted regression (MGWR) model, the hypothesis was tested using Henan Province in China as a case study. The results indicated that the phenomenon of STS was observed extensively across the study region, with a 59% geographical overlap between the high-value area of STS and the low-value area of urban-rural interaction. Three distinct sub-types of STS regions were identified: shrinking regions along geographical borders, shrinking regions adjacent to metropolitan areas, and shrinking regions in ecologically fragile areas. The factors influencing STS demonstrated spatial heterogeneity and multi-scale characteristics. The findings will improve our understanding of urban shrinkage from a multi-level perspective and offer policy makers guidance for the sustainable development of small towns based on local conditions.

## Introduction

The 21^st^ century has been an era of demographic changes [1]. The current phenomena of urban shrinkage does not fit with the permanent growth paradigm of cities, and a new form of development has been initiated, i.e., a non-growth-oriented development [2]. Urban shrinking has become a significant issue in the world, affecting not only large and medium-sized cities [3], but also small towns [4]. However, in the context of globalization and the “Urban Age”, researchers are now increasingly focused on the shrinkage and planning response of large and medium-sized cities, with less concern expressed toward small towns [5, 6]. The significance of small towns in bridging the gap between urban and rural areas [7], particularly within the context of global rural decline amidst worldwide efforts to promote urbanization and industrial development, should not be underestimated [8, 9]. However, they are facing several development problems, such as not being attractive to the urban population, a low level of rural population migration, and a low rate of local urbanization [10]. Receding economic growth and a declining population in small towns are pivotal factors contributing to the erosion of incomes and the exacerbation of poverty in rural communities [11]. Our work has great theoretical and practical significance, particularly for the governance, transformation, and rebirth of shrinking towns [12].

China’s rapid urbanization has resulted in the concentration of resources, such as labor forces and land resources, from rural to urban areas. However, small towns in China have been unable to reap the benefits of urbanization due to a lack of skilled workers, limited financing channels, loss of labor force, insufficient infrastructure development, mono-industrial structure, weak management practices, and restricted jurisdiction [13], which has inevitably resulted in the decline of small towns [14]. Currently, one-third of China’s small towns are witnessing a decline in population density and experiencing severe urban decay [15], necessitating heightened attention. Fortunately, the Chinese government is effectively implementing the rural revitalization strategy to address the decline in rural areas. This comprehensive strategy aims to establish a symbiotic relationship between urban and rural regions, fostering integrated development and reshaping their dynamics. Small towns play a pivotal role in this strategy as they serve as a crucial link connecting urban and rural areas, acting as vital nodes within the overall rural territory or residential system. [16]. However, STS is a complex process with various causes that are strongly correlated with the geographic environment. A crucial initial step towards addressing this issue from a scientific standpoint involves the identification of distinct types of shrinkage.

The remainder of this paper is structured as follows. A concise review of the relevant literature is provided in the literature review section. Building upon prior research, the methodology applied and data used for the analysis are introduced in the section of materials and methods. The results section presents the obtained results, while a comprehensive discussion and conclusion are respectively presented in the discussion and conclusion sections.

## Literature review

### What is a small town and what is its role in the rural-urban system?

There is no universally agreed upon definition of what constitutes a small town [17]. Traditionally, the definition of a town encompasses three primary methodologies: the morphological approach, the functional approach, and the administrative approach. However, previous studies have predominantly focused on employing the morphological approach to delineate population density through polygons. However, such categorization is excessively inclusive and inevitably obscures the substantial variations within and across regions. [18], consequently impeding comparability between countries. In China, small towns (referred to as *Xiangzhen* in Chinese) are classified based on their administrative management unit, which corresponds to the fourth level of regional administration hierarchy: provincial administrative region, prefecture-level administrative region, county administrative region, and township administrative region from top to button. The spatial structure of small towns in China comprises two components: an urbanized area, similar to that found in developed countries, and an area covering a more rural population and agricultural land. Small towns in developed countries generally do not contain a significant rural population or areas of agricultural land, with the land being mainly urbanized areas [19]. This study specifically focused on the urbanized area of small towns in China, which can be considered consistent across the country and comparable with small towns in developed countries.

We have adopted the functional approach to define small towns, because of their key role in the urban-rural relationship, which has evolved from an urban-rural dichotomy to urban-rural integration. The acceleration in the historical urbanization rates and its associated social problems provide the context for the emergence of an urban-rural dichotomy that was particularly prominent during the late 19^th^ century. Over time, this dichotomy gave rise to two contrasting schools of thought. At one end of the spectrum was the anti-urban view which idealized and regretted the disappearance of rural life; at the other end was the pro-urban view which considered urbanization as the engine of progress, innovation, and modernization. The concept of urban-rural relationships has emerged as a way of challenging this longstanding and persistent dichotomy and promoting an integrated conception of cities and the countryside based on both their spatial and functional interdependencies. It was not until the 1960s, when the spatial linkages between urban and rural areas became a common concern that urban analysts shifted their focus from the city to encompass the broader city-region. In his vision for spatial development, Melvin Webber challenged the notion that “urban and rural areas represent a dichotomy that must be clearly manifested in the physical and spatial structure of cities, with orderliness contingent upon strict boundaries” [20]. Over time, this symbiosis has undergone transformations; however, towns can still be regarded as crucial instruments in rural development, not only in peripheral regions but also in the proximity of urban areas. Towns serve as convergence points for rural activities and often offer organizational advantages [21]. The linkages between urban centers and the countryside, encompassing the intricate dynamics of human mobility, trade flows, financial investments, and various socio-economic interactions, exert a pivotal influence on the transformative processes shaping rural and urban landscapes.

**Fig 1:**
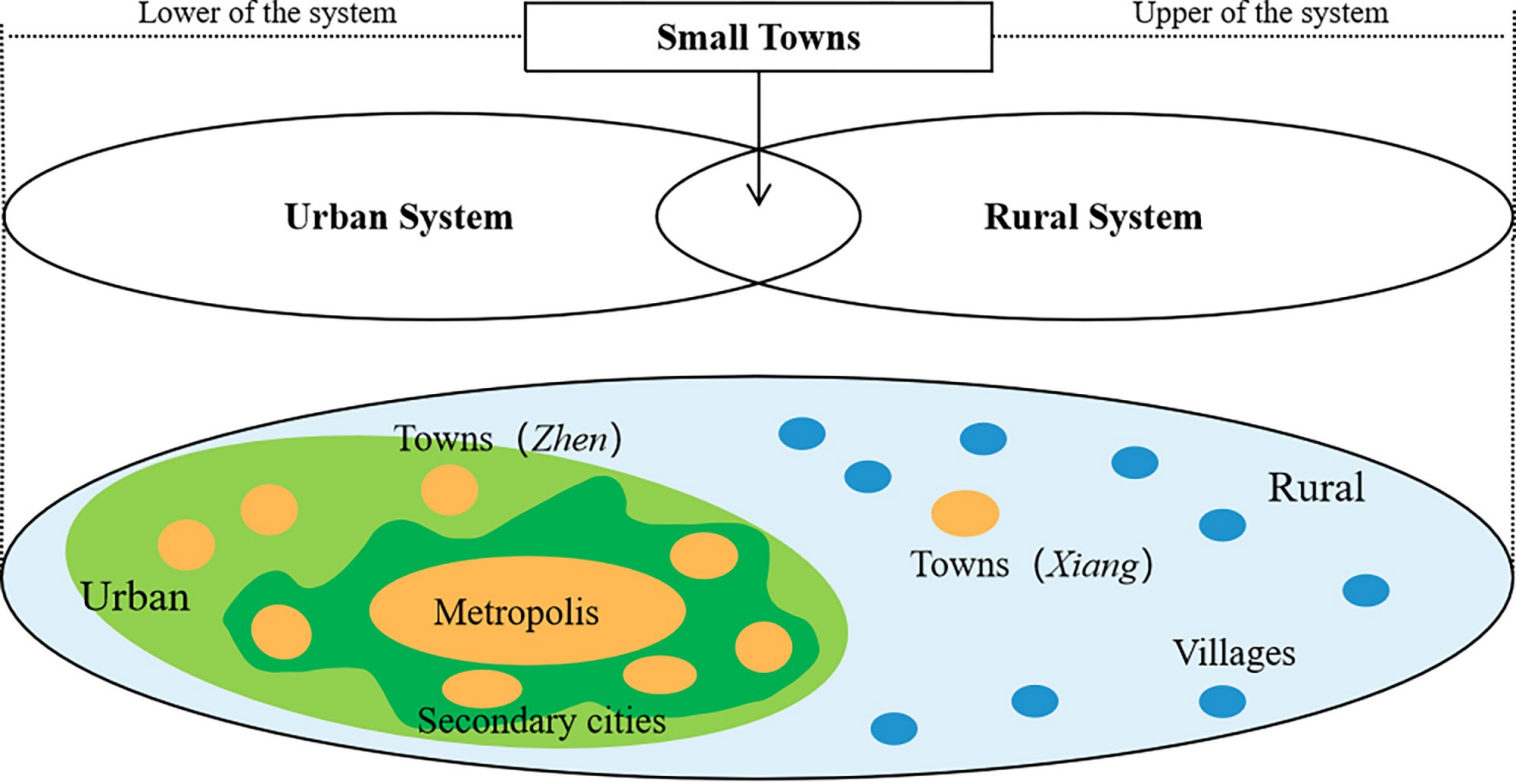
The position of small towns within the rural-urban system.

From the perspective of an urban and rural territorial system, small towns serve a dual purpose by functioning as both an extension and hinterland of cities, thereby representing the lower layer of the urban territory system. Conversely, they constitute the upper layer of the rural territory system, exercising jurisdiction over vast rural areas. Small towns possess significant functions that warrant their allocation. Firstly, the economic development of small towns plays a vital role in facilitating urban-rural interaction. Secondly, small towns serve as crucial hubs for farmers to exchange and distribute their agricultural products. As a transitional carrier bridging urban and rural areas, small towns exhibit remarkable sensitivity towards changes in urban-rural relationships. Their rise or decline serves as a concentrated manifestation of regional production flows and shifts in urbanization patterns [22]. We have created a schematic map (Fig 1) to illustrate the pivotal role of small towns within the urban-rural system. Small towns function as “urban centers” in the rural territory and constitute an integral part of the expanded urban settlement system. They occupy a crucial position within the growing economic network and serve as a vital link in the rural-urban system, akin to small towns found in numerous developed countries [23]. Consequently, this perspective aligns more consistently and comparably with small towns observed globally.

### Why do small towns shrink? The phenomenon and mechanism

**Fig 2:**
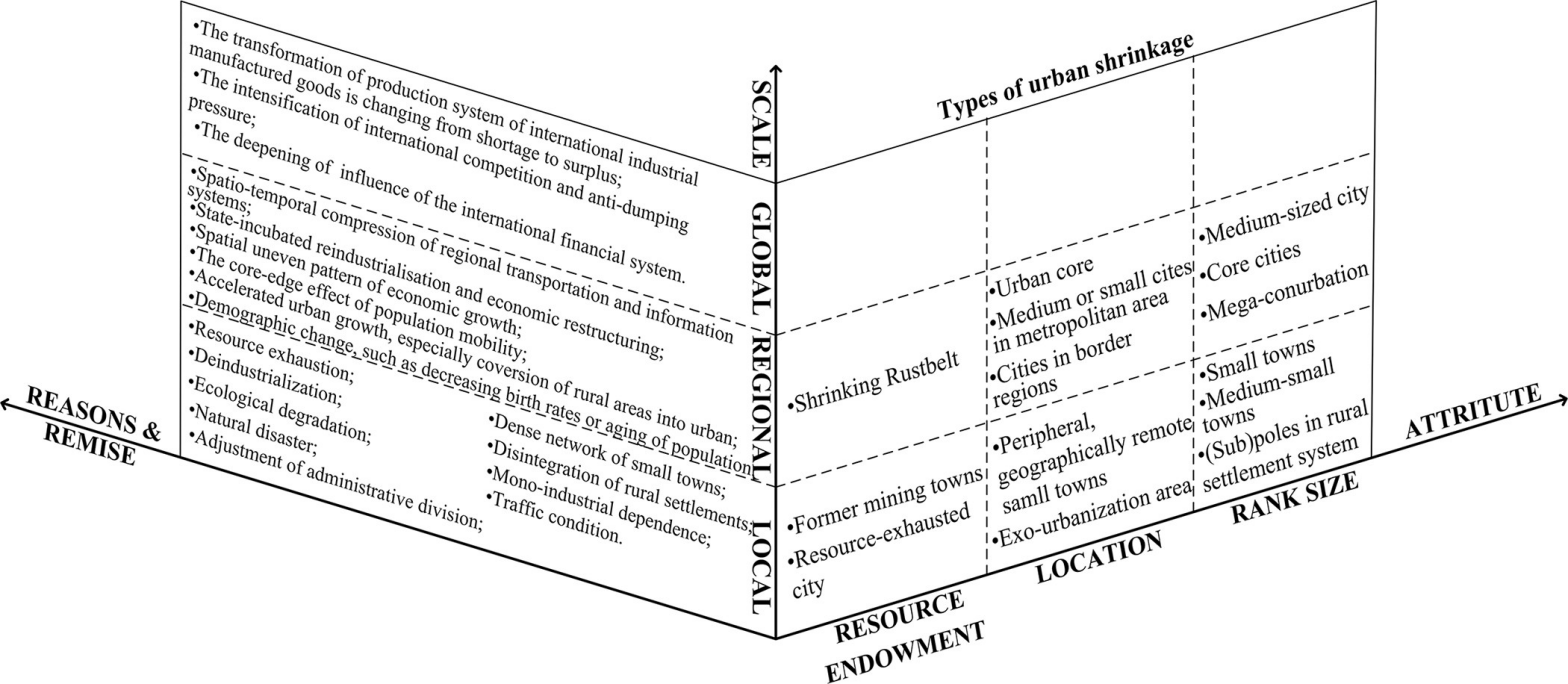
The framework applied to understand the phenomenon and mechanism of urban shrinkage.

As an integral component of the urban regional system, it is crucial to comprehend the spatial contraction and driving mechanisms underlying small town shrinkage from a broader perspective. Typical symptoms of shrinking urban towns include population decline and economic downturn [24, 25]. Studies have revealed that city and town shrinkage exhibits hierarchical differences, with high-level cities experiencing widespread and slight shrinkage, while low-level towns suffer the most severe and persistent shrinkage [26]. The drivers of STS are complex and diverse, often resulting from a combination of globalization and localization factors that contribute to migration from small towns, economic deterioration, industrial collapse, and loss of employment opportunities [27]. The processes causing urban shrinkage vary considerably depending on the national, regional, and local contexts. We reclassified spatial phenomenon and the mechanism of urban shrinkage from the three dimensions of scale, attributes, and reasons and premise (Fig 2). At the global scale, main factors leading to urban shrinkage include the transformation of the production system of international industrial manufactured goods, the intensification of international competition and anti-dumping pressure, and the increasing influence of the international financial system [28], such as experiences observed in the Rustbelt cities of the United States [29]. At the regional scale, according to the location or rank size of cities, there are distinct types of shrinking cities, such as urban core, core cities, medium, or small cites in metropolitan areas and mega-conurbations. Reasons for this phenomenon vary accordingly. For instance growing city regions have witnessed core area shrinkages due to suburbanization being one major contributing factor [30]. Shrinkage in small towns predominantly occur at local scales considering the resource endowment of cities, economic decline in old, industrialized cities due to resource exhaustion has resulted in shrinking cities, such as Glasgow, Liverpool, and Manchester in Europe, and Detroit, Pittsburg, Cleveland, and Youngstown in the United States. Many mining towns have experienced periods of growth and shrinkage, mirroring the ebbs and flows of international mineral markets, which determine the fortunes of the dominant mining corporation upon which each of these towns are heavily dependent. This dependence on one main industry produces a parallel development in the fluctuations of both the workforce and population [31]. Moreover, depending on their location or rank size of the towns, the functions of small towns that are peripheral, geographically remote, or (sub)poles in the rural settlement system may be undermined by changes in economic connections and mobility patterns as well as by administrative adjustments, disintegration of rural settlements, dense networks of small towns, and inadequate transportation infrastructure resulting from the state’s withdrawal from rural areas [4]. The constraints on the development of small towns are intricate due to the increasing level of interference as the scale of settlement decreases. Factors such as natural resource endowment, population density, market accessibility, location conditions, and political and economic structures can significantly impact the development of small towns [32, 33]. However, the driving mechanisms of STS in China and the West differ due to the varying stages of economic development. For example, developed countries are mainly affected by deindustrialization under the background of globalization, while in China, a more prominent role is played by a low-level industrial structure [34]. At the regional scale, the function of a small town within its regional context shapes its development. Many small towns serve as ‘urban centers’ for their rural surroundings, and their shrinkage occurs in the same way as unsuccessful cities in developed countries. It has long been acknowledged that small towns are a key element of Europe’s urban structure, both historically and in the modern era, making them an integral part of the continent’s urban fabric [17]. Consequently, these small towns often have limited opportunities to shape their own destinies.

In summary, previous research tends to categorize small towns as part of the urban territory system without acknowledging their crucial role as a “bridge” in RUI. It is imperative to recognize that small towns are not isolated entities. While some studies have acknowledged the bridging function of small towns in theory, empirical research has not sufficiently explored this function within the rural territory system. Additionally, it is of significance to develop the typology of STS to interpret the development paths of the large number and diverse types of small towns. From the functional perspective, small towns serve as both bases for diffusing urban civilization and centers for organizing the development of rural territories [35]. However, most previous studies have examined small towns solely as endpoints in the urban system neglecting their potential shrinkage from an RUI perspective. To investigate the relationship between RUI and STS, we must first understand that China is undergoing rapid urbanization with predominantly rural-to-urban flows in RUI. A high value of RUI indicates a substantial concentration of rural elements flowing into urban areas, while a low value signifies a limited concentration. Based on this premise, we propose the hypothesis that there is a negative relationship between RUI and STS. To explore this relationship, we aim to address the following questions:

1. Is there a negative correlation between the degree of STS and the intensity of RUI, and how is this relationship manifested geographically?
2. If such a negative correlation exists, what are the primary factors contributing to STS? Are there regional variations in the factors affecting the shrinkage?

## Materials and methods

### Study area

**Fig 3:**
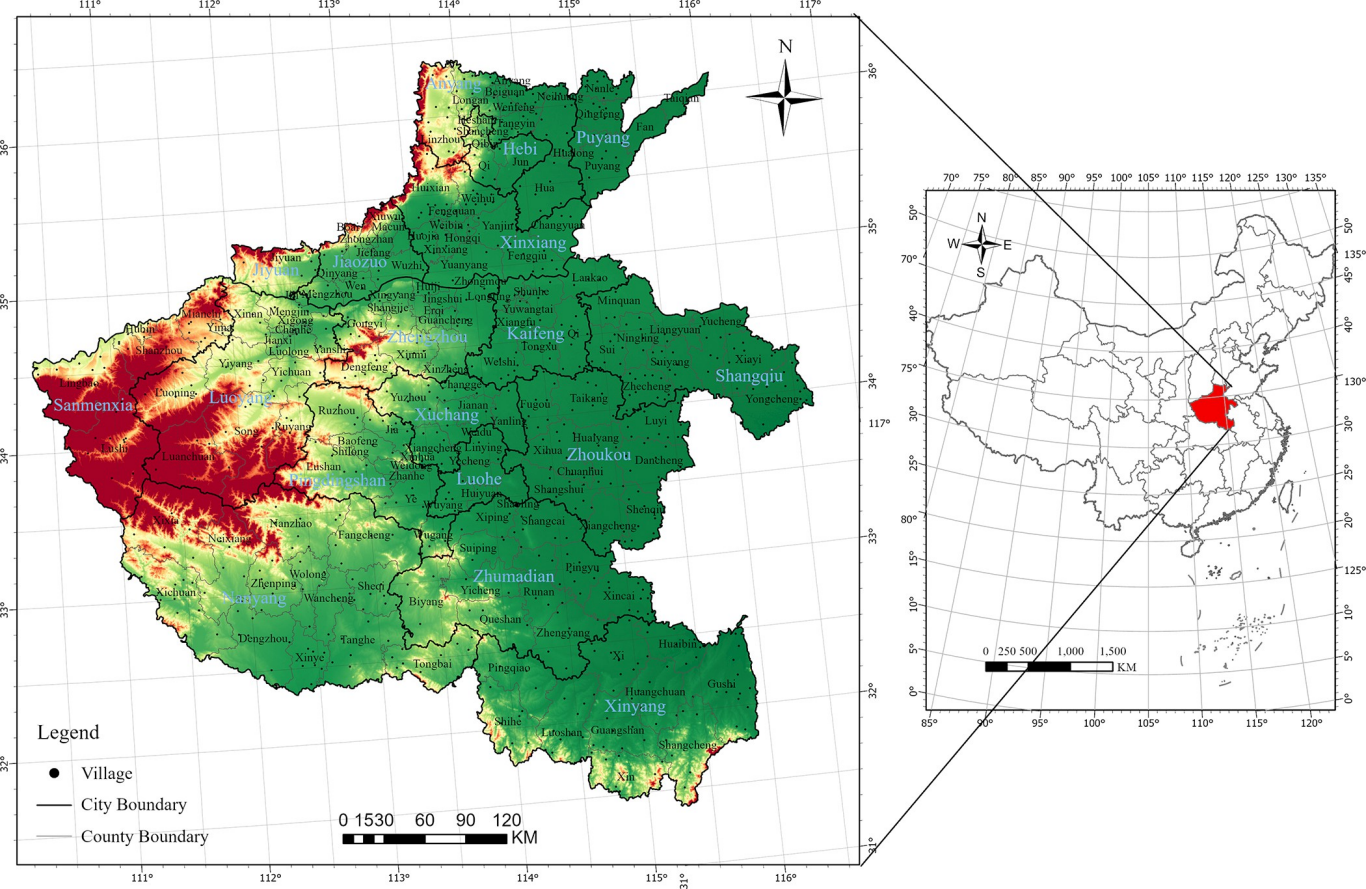
The location of the study area.

Henan Province, located in central China, is recognized as a traditional agricultural province (Fig 3), possessing 6.871 million ha of arable land, which ranks second among all provinces in China. As of 2021, the permanent resident population in Henan Province reached 98.83 million. Among them, 43.04 million people resided in small towns, accounting for 43.55% of the permanent residential population. Moreover, Henan Province has long been acknowledged as a major labor exporting province, with 115.33 million people registered in small towns by 2021, and a high outflow rate that accounted for 62.68% of the population. For our study, we specifically focused on 1,677 towns in Henan Province. However, it should be noted that there were originally 1,791 towns recorded by the end of the year 2020 within this region’s administrative boundaries. We excluded a total of 72 towns from our analysis due to their undergoing significant administrative readjustments during the study period which resulted in substantial changes to both their populations and built-up areas rendering them incomparable to other towns under investigation. Furthermore, 42 additional towns falling under an area commonly referred to as “Chengguan Town” were also eliminated from our research scope since this name is frequently used to denote county government seats that do not fall within our designated research area.

### Variable selection and data sources

#### Calculation of the intensity of RUI

The RUI evaluation index system is a comprehensive system consisting of a set of interrelated and independent index factors that can be measured quantitatively [36]. It encompasses indicators from four dimensions: rural-urban spatial connection, economic ties, social integration, and functional identification. To calculate the intensity of RUI, we used a comprehensive index method that had the principles of being scientific, comprehensive, and available. We obtained data from the Henan Statistical Yearbook (HSY) in 2021 and City Statistical Yearbook (CSY) in Henan province in 2021 and made calculations at the county scale. Table 1 shows the indicators used for each dimension.

**Table 1:** Index system used to calculate the intensity of RUI.

Dimension	Index layer	Criteria layer	Data sources
Rural-urban spatial connection (RUSP)	Spatial agglomeration (SA)	X_1_: Urbanization rate (%)	HSY
	Transportation convenience (TC)	X_2_: Accessibility from administrative location of towns to county	Openstreet Map
	Information degree (ID)	X_3_: Per capita post and telecommunications business volume (ten thousand yuan)	HSY&CSY
Rural-urban economic ties (RUET)	Economic aggregation (EA)	X_4_: GDP per capita (Yuan)	HSY
	Industrial structure (IS)	X_5_: Proportion of non-agricultural industry output value (%)	HSY
	Employment structure (ES)	X_6_: Proportion of employees in non-agricultural industry (%)	HSY&CSY
	Income gap (IG)	X_7_: Per capita income ratio of urban and rural residents (%)	HSY&CSY
		X_8_: Wage income ratio of urban and rural residents (%)	HSY&CSY
Rural-urban social integration (RUSI)	Educational services (ES)	X_9_: Proportion of the number of teachers and students in compulsory education (%)	HSY&CSY
	Medical services (MS)	X_10_: Hospital beds per 10,000 people	HSY
		X_11_: Staff per 10,000 people	HSY
	Living allowances (LA)	X_12_: Proportion of the number of urban and rural residents registered for endowment insurance (%)	HSY&CSY
		X_13_: Proportion of the number of urban and rural residents enjoying minimum living allowances (%)	HSY&CSY
	Consumption gap (CG)	X_14_: The per capita consumption ratio of urban and rural households (%)	HSY&CSY
		X_15_: Ratio of the urban-rural Engel coefficient (%)	HSY&CSY
Rural-urban functional identification (RUFI)	Functional identification of the urban system (FIUS)	X_16_: GDP per land in the built-up area of the county (100 million yuan/square kilometer)	HSY
		X_17_: Construction land area per capita (square meter/person)	HSY
	Functional identification of the rural system (FIRS)	X_18_: Entropy of average land yield of grain	HSY&CSY
		X_19_: Agricultural mechanization level	HSY&CSY

#### Calculation of the degree of STS

Various definitions have been employed to delineate urban shrinkage, although they commonly highlight population decline as the primary symptom [37]. There are differences between countries in terms of administrative classification criteria and population size. Our study on STS was approached from a functional perspective, and thus we did not give a standard threshold of STS. Instead, we solely focused on delineating the spatial pattern problem based on the degree of shrinkage. The study period spanned from 2015 to 2020. The degree of STS was calculated from two dimensions: changes in total population and economic development. The degree of total population shrinkage was calculated by two indicators, namely, the average annual growth rate of the residential population and the average annual growth rate of the residential population living in the built-up area. The degree of economic development shrinkage was calculated by three indicators, namely, the average annual growth rate of industrial enterprises, the average annual growth rate of enterprises with an annual main business income of 20 million yuan or more, and the average annual growth rate of the number of general stores or supermarkets with a business area of 50 m^2^ or more. The research data were obtained from the China Statistical Yearbook (Volume of Township) from 2016 to 2021. Table 2 shows the variables used for each dimension.

**Table 2:** Index system used to calculate the degree of STS.

Dimension	Variables	Data sources
Population change	S_1_: The average annual growth rate of the residential population(%)	China Statistical Yearbook
	S_2_: The average annual growth rate of the residential population living in the built-up area(%)	China Statistical Yearbook
Economic development	S_3:_The average annual growth rate of industrial enterprises(%)	China Statistical Yearbook
	S_4:_The average annual growth rate of the number of general stores or supermarkets with a business area of 50 m^2^ or more(%)	China Statistical Yearbook

### Main methods

#### Entropy weight TOPSIS

The entropy weight TOPSIS method represents an enhancement of the conventional TOPSIS evaluation approach. In this method, the weight of the evaluation indices are determined through the entropy weight method, and then the TOPSIS method uses technology to approximate the ideal solution. The entropy weight method is used to objectively determine the weight according to the information provided by each evaluation index. Because the entropy is a weighted number, it can objectively reflect the importance of an index in the index system when making decisions. The details of the operation are presented in Martin et al. [38].

#### Multiscale geographical weighted regression (MGWR)

Different spatial, economic, social, and subjectivity functions may have various urban-rural connections which may result in a spatial effect on STS at changeable scales, which means spatial heterogeneity and homogeneity may coexist. In this study, an ordinary least squares (OLS) regression was first applied as a preliminary test of the correlation between STS and explanatory variables and the key results were selected for further analysis by GWR and MGWR [39, 40].

*1*. *The OLS regression*. The traditional hedonic model could be expressed as an OLS regression, which means for each observation of STS *y*_*i*_:

$$y_{i}=\beta_{0}+\sum \beta_{i}x_{i}+\varepsilon_{i}$$ (1)

where *β*_0_ is the intercept, x_i_ represents the independent variable, *β*_*i*_ is the corresponding coefficient, and *ε* is the error.

*2*. *The GWR*. Compared to the OLS regression model, the GWR model strengthened the expression of spatial heterogeneity by adding space-varying parameters according to the coordinates of each observation (*u*_*i*_, *v*_*i*_). The GWR equation for STS *y*_*i*_ could be written as:

$$y_{i}=\beta_{0}(u_{i},v_{i})+\sum_{k}\beta_{k}(u_{i},v_{i})x_{ik}+\varepsilon_{i}$$ (2)

where *β*_0_(*u*_*i*_, *v*_*i*_) and *β*_*k*_(*u*_*i*_, *v*_*i*_) are the intercept and the coefficient of local variable *k* at location *i*, respectively, and *x*_*ik*_ is the *k* variable at location *i*.

A GWR using a single bandwidth is not capable of expressing such features, while an MGWR could overcome these problems by assigning specific bandwidths for each variable based on an iteration. The MGWR could be written as:

$$y_{i}=\beta_{bw_{0}}(u_{i},v_{i})+\sum_{k}\beta_{bw_{k}}(u_{i},v_{i})x_{ik}+\varepsilon_{i}$$ (3)

The OLS, GWR, and MGWR models could be applied directly via the MGWR software [41].

## Results

### Spatial distribution and characteristics of STS

**Fig 4:**
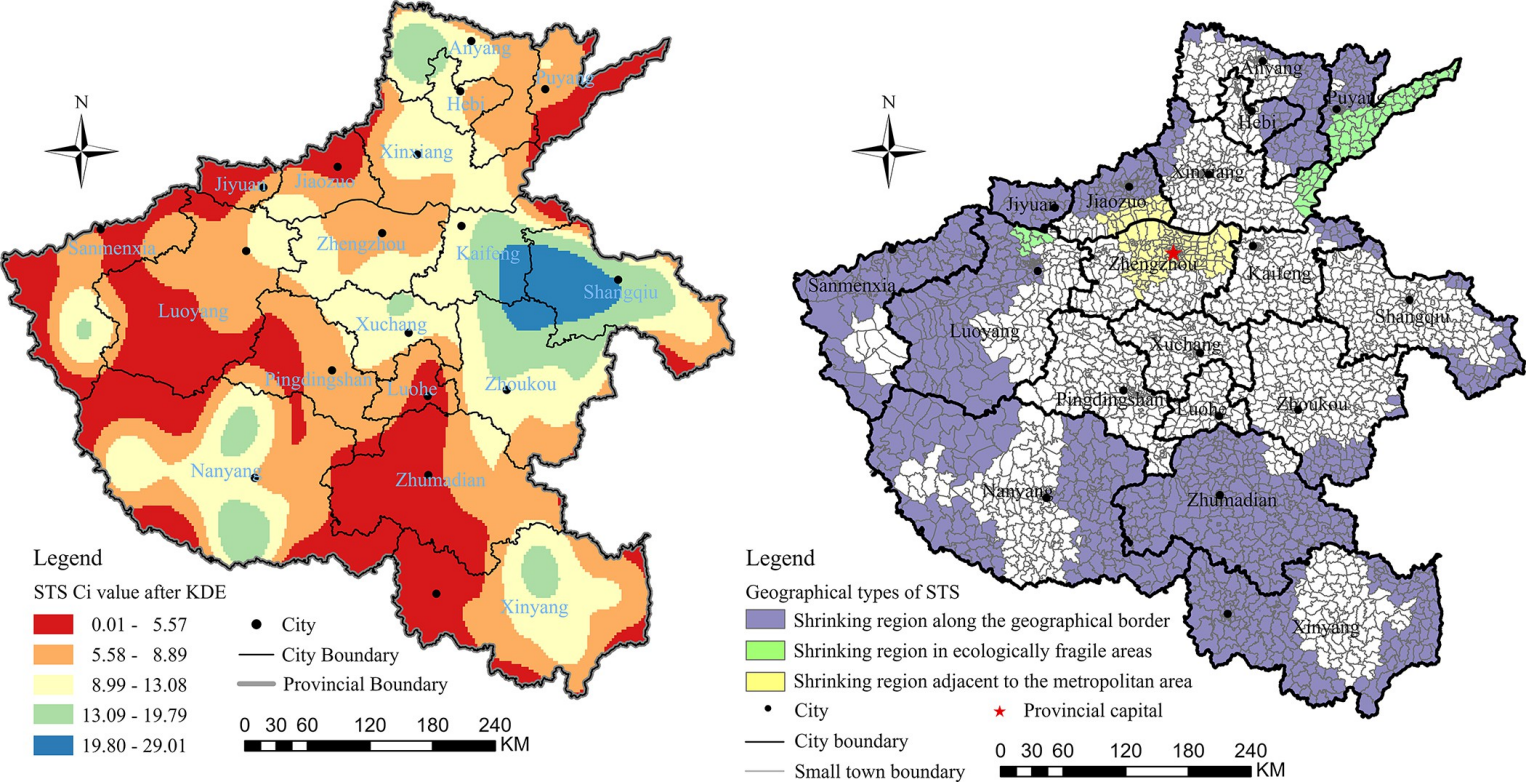
The spatial pattern of STS in Henan province (a) and geographical types of STS (b).

During the study period from 2015 to 2020, it was found that 878 of 1,677 small towns in Henan Province experienced a negative growth rate in terms of their residential population, accounting for 52.36% of the total number. Furthermore, the number of industrial enterprises decreased in 1,174 small towns (70% of all), and the number of industrial enterprises with an annual main business income of 20 million yuan or more showed negative growth in 630 small towns (37.67% of all). However, only 215 small towns (12.82% of all) experienced a decline in the number of general stores or supermarkets, which play a crucial role in facilitating daily living functions. daily living functions. The entropy weight TOPSIS method was employed to calculate the degree of STS in Henan Province. The results were then visualized using the ArcGIS Pro platform after conducting a kernel density estimation (KDE) based on the C_*i*_ values of the degree of STS and its subsystems (Fig 4A).

The results indicated that the characteristics of STS could be attributed to geographical marginality, with areas exhibiting a higher degree of shrinking primarily located in the inter-provincial border regions, especially along the mountainous areas from northwest to south in Henan Province, including the Taihang, Fu Niu, and Dabie mountains. Another significant shrinking region was identified adjacent to the metropolitan area, specifically Zhengzhou City, the capital of Henan Province. This phenomenon was attributed to the siphon effect of the metropolitan area on the surrounding towns, resulting in the migration of population and economic elements towards the core city. Consequently, the spatial pattern of the small towns adjacent to Zhengzhou City displayed moderate shrinkage. Finally, typical shrinking regions were also found in ecologically fragile areas, such as the Yellow River beach area that extends from Baihe, Mengjin County, Luoyang City to Zhangzhuang, Taizian County, and Puyang City. The beach area has long been associated with poor living and production conditions, as well as relatively weak economic development, and certain areas are considered key poverty zones in both Henan Province and China overall. In summary, the study identified three sub-types of STS regions, namely the shrinking region along the geographical border, the shrinking region adjacent to the metropolitan areas, and the shrinking region in ecologically fragile areas (Fig 4B).

### The spatial pattern of RUI intensity and its geographical relationship with STS

**Fig 5:**
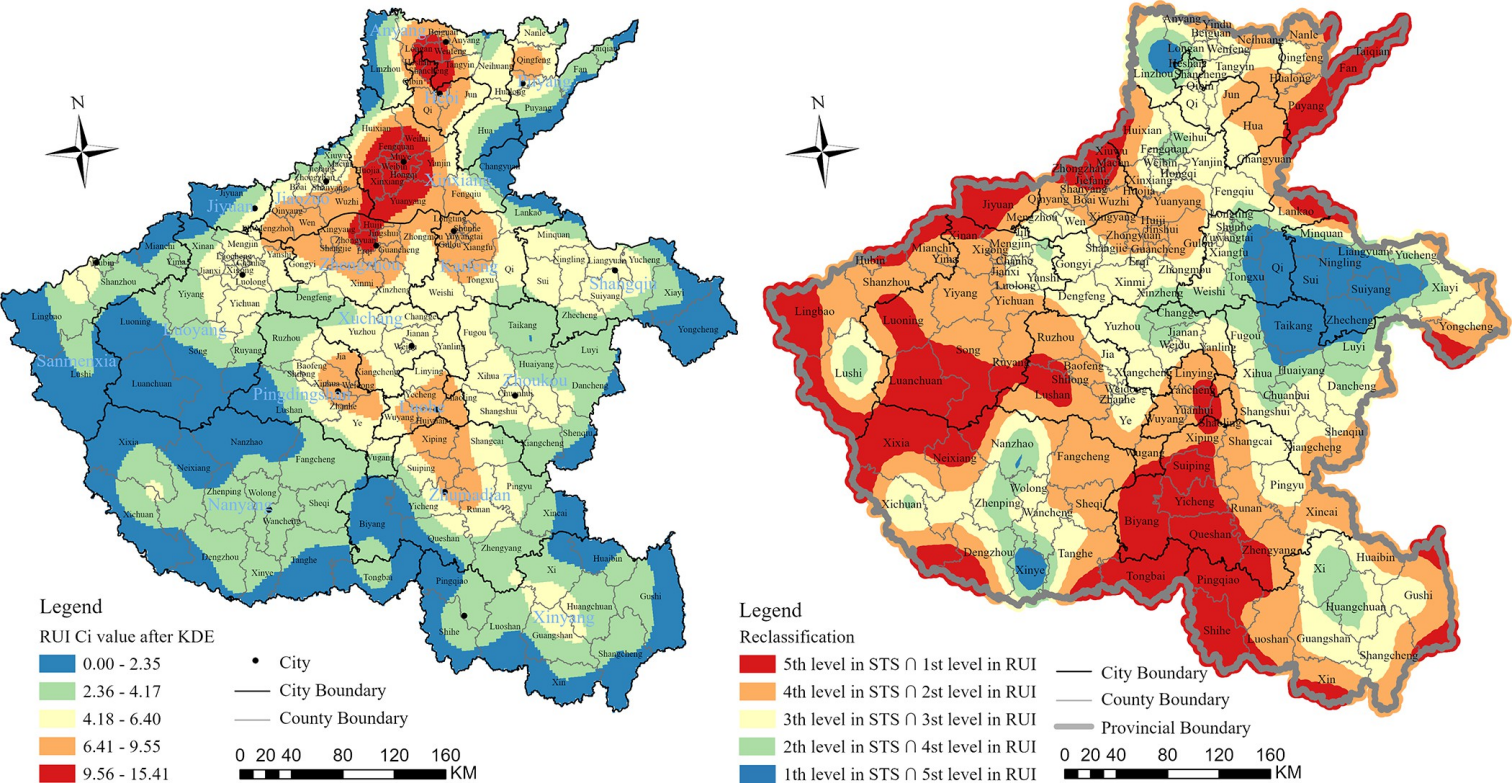
The spatial pattern of the intensity of RUI (a) and the pattern of its intersection with the degree of STS (b).

To measure the intensity of RUI in Henan province, we used the entropy-weighted TOPSIS method to obtain C_*i*_ values, which were then classified into five categories based on the natural breakpoints classification (NBC) method. The spatial pattern of RUI intensity is presented in Fig 5A, which was created using ArcGIS Pro software. Our analysis revealed that RUI exhibited a clear “core-periphery” structure, with the highest intensity regions concentrated along the Zhengzhou-Xinxiang-Anyang urban corridor, which was close to the capital metropolitan area. The intensity of RUI gradually decreased from the core to the periphery regions. Furthermore, we found that the edge of the provincial boundary exhibited a lower RUI intensity, reflecting its geographical marginality. We also identified the three indicators with the highest weight coefficients based on the entropy-weighted TOPSIS method: the value of agricultural mechanization (X_19_), which reflects the functional identification of the rural system, had a weight coefficient of 19.09%; the urbanization rate (X_1_), which reflects spatial agglomeration, had a weight coefficient of 15.79%; and the proportion of employees in non-agricultural industry, which reflects educational services, had a weight coefficient of 14.16%.

To explore the spatial connection between the intensity of RUI and the degree of STS, the overlay analysis tool was used to intersect Figs 4A and 5a. First, the KDE maps of STS and RUI were raster-transformed in ArcGIS Pro software to obtain the point distribution maps of the two transformations, respectively. Second, the point distribution maps were divided into five levels according to the NBC, and intersected using the intersect function in the overlay analysis tool boxes in the software, with the rule that the fifth level of the STS intersected with the first level of the RUI, and so forth (Fig 5B). The first two levels were selected as the study subjects, and the percentage of study subjects was calculated according to the Monte Carlo algorithm. First, a coordinate system was established according to Fig 3B, with the X-axis connected to the bottommost boundary of the map and the Y-axis connected to the leftmost boundary of the map. The scale interval of the X and Y axes of the coordinate system was [0, 1], so that the whole picture was placed in a 1 × 1 square. Next, the MATLAB software was used to program the number of points to be randomly scattered within the interval of X and Y axes, and the number of points was 1000. The percentage of each color was derived based on the number of dots within each color. Finally, it was calculated that the first level, i.e., the red-covered regions in Fig 4B, accounted for 29% (48,430 km^2^) of the total area, and the second level, i.e., the orange-covered regions in the map, accounted for 30% (50,100 km^2^) of the total area. The summed percentage was 59% (98,530 km^2^), which means there was a 59% geographical overlap between the high-value-degree of STS and low-value-intensity of RUI.

### Spatial heterogeneity analysis of the driving factors

The spatial heterogeneity of the relationship between the degree of STS and intensity of RUI was investigated at the county scale. Firstly, the mean *Ci* values of STS in each county were taken as the dependent variable (Y), while the factors influencing the intensity of RUI were taken as the independent variables (X); Subsequently, OLS, GWR, and MGWR were calculated based on Eqs 1 to 3 using MGWR and ArcGIS Pro software. The R^2^ values from the OLS, GWR, and MGWR were 0.995, 0.995, and 0.998, respectively indicating fitting degree of 99.8% for the MGWR model. This confirms that the mechanism governing spatial heterogeneity in STS can be effectively verified using the MGWR model.

### Model comparison

During the application of MGWR, both the GWR and MGWR results were generated by the MGWR software with the interval bandwidth searching method (Table 3). The results showed that although both GWR and MGWR demonstrated significant enhancements compared to the OLS results, the latter displayed a higher level of model fit.

**Table 3:** Types of interaction detectors.

	R^2^	Adj. R2	AICc	Sigma estimate	Residual sum of squares (RSS)
OLS	0.995	0.994	-1044.344	一	一
GWR	0.995	0.994	-337.939	0.072	0.671
MGWR	0.998	0.997	-371.687	0.058	0.369

### Estimates of MGWR parameters

Compared to the bandwidth value of 157 displayed by the GWR model, MGWR assumed that the bandwidth ranged from 44 to 156 (Table 4). The results showed that ten variables, namely X_1_, X_4_, X_6_, X_8_, X_10_, X_11_, X_12_, X_16_, X_18_, and X_19_ exhibited a global scale, while the remaining variables demonstrated a local scale effect. This suggests minimal spatial heterogeneity and indicates that the influence of these aforementioned variables on STS is consistent across all regions. Concerning the global variables, with the exception of X_18_ which showed no significant impact on STS; all others had a negative impact. In terms of the local variables, except for X_9_ and X_17_ which did not exhibit any significant impact on STS; all other variables had a negative impact.

**Table 4:** Summary statistics for MGWR parameter estimates.

Variable	Bandwidth	Coefficients				
		Mean	STD	Min	Median	Max
Intercept	156	-0.002	0.001	-0.004	-0.002	-0.002
X_1_	156	-0.472	0.002	-0.477	-0.472	-0.470
X_2_	44	-0.016	0.016	-0.044	-0.021	0.014
X_3_	109	-0.012	0.004	-0.022	-0.011	-0.006
X_4_	156	-0.026	0.01	-0.027	-0.026	-0.023
X_5_	44	-0.047	0.022	-0.008	-0.049	-0.005
X_6_	156	-0.378	0.001	-0.379	-0.378	-0.377
X_7_	59	-0.120	0.039	-0.186	-0.107	-0.075
X_8_	156	-0.050	0.001	-0.052	-0.050	-0.049
X_9_	78	0.020	0.024	-0.019	0.018	0.053
X_10_	152	0.000	0.002	-0.004	0.000	0.003
X_11_	156	-0.004	0.001	-0.006	-0.003	-0.003
X_12_	156	0.015	0.000	0.014	0.015	0.015
X_13_	97	-0.031	0.016	-0.069	-0.022	-0.020
X_14_	156	-0.082	0.001	-0.084	-0.082	-0.080
X_15_	91	-0.027	0.008	-0.045	-0.025	-0.012
X_16_	156	0.012	0.000	0.012	0.012	0.013
X_17_	130	0.008	0.003	0.000	0.008	0.012
X_18_	156	0.010	0.000	0.009	0.010	0.010
X_19_	156	-0.544	0.001	-0.547	-0.544	-0.542

### The spatial heterogeneity of STS

**Fig 6:**
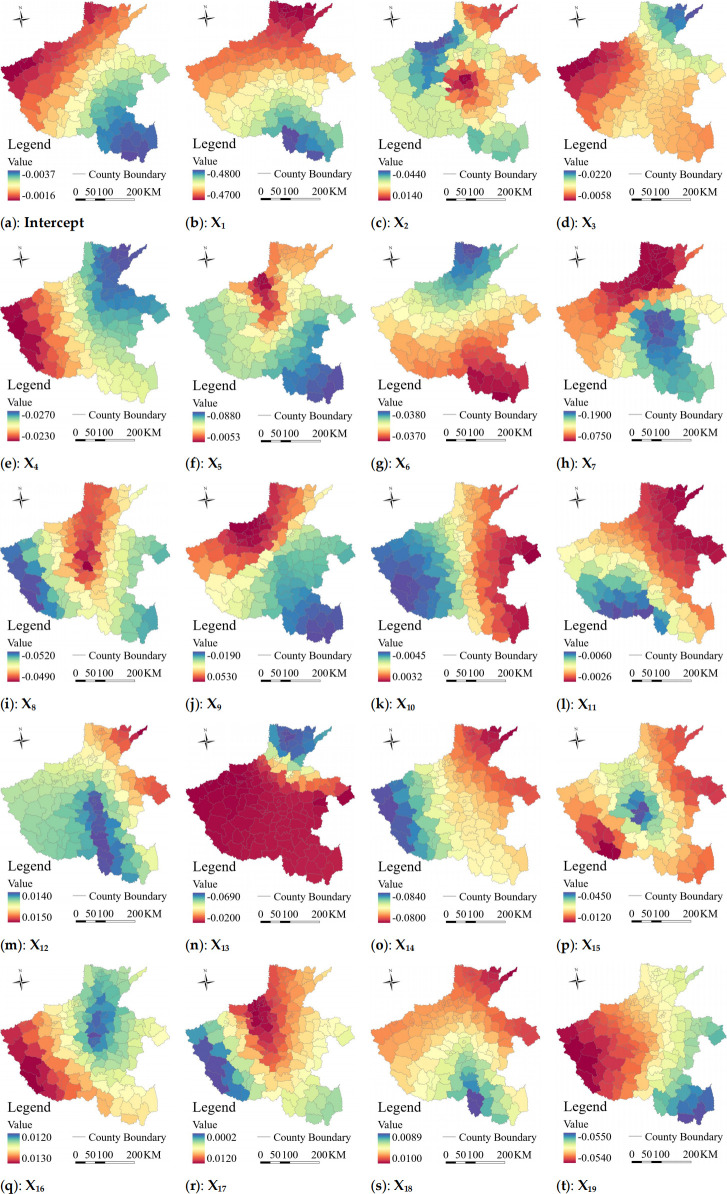
The impact of the spatial heterogeneity of the factors of the RUI system on STS.

The MGWR results revealed spatially varying scale characteristics of all variable coefficients, accompanied by the acquisition of their statistical p-values, thereby providing more precise insights into the co-occurrence of spatial heterogeneity. Notably, the marginal mountainous areas at a global scale exhibited significant influence from the intensity of RUI on STS (Fig 6A). Specifically, four dimensions of the intensity of RUI were considered.

#### 1. Rural-urban spatial connection (RUSP)

The three indicators in the RUSP dimension exhibited a negative correlation, indicating that higher intensity of RUSP was associated with lower degrees of STS, which aligns with the hypothesis of this study. Furthermore, there existed spatial heterogeneity, with transportation convenience displaying localized characteristics. Specifically, the shrinking region along the geographical border was primarily influenced by the rural-urban information degree, the shrinking region in the ecologically fragile area was mainly influenced by the two factors of rural-urban spatial agglomeration and transportation convenience, and the shrinking region adjacent to the metropolitan area was predominantly influenced by the rural-urban transportation convenience. Notably, due to its localized nature, rural-urban transportation convenience had a significant local explanatory effect on both ecologically fragile areas and the region adjacent to the metropolitan area (Fig 6B–6D).

The underlying reasons contributing to this phenomenon can be summarized as follows. 1. Efficient transportation infrastructure facilitates seamless mobility between urban and rural areas, fosters the flow of goods and services, attracts increased business activities and investments, and enables enhanced economic exchanges between small towns and the external world. Moreover, it contributes to more optimal resource allocation encompassing healthcare, education, and other social services while augmenting the allure of small towns, all of which could mitigate the issue of population decline in small towns. 2. Robust information connectivity enables small towns to swiftly access valuable information and knowledge, thereby facilitating local enterprises and residents in seizing novel business opportunities and fostering development. Moreover, it promotes the dissemination and application of technology, enhances social and cultural exchanges, as well as facilitates garnering attention and support from governmental bodies and other organizations. This holds immense significance in augmenting production efficiency and competitiveness within small towns.

#### 2. Rural-urban economic ties (RUET)

The five indicators in the RUET dimension exhibited a negative correlation, indicating that as the intensity of RUET increased, the degree of STS decreased., which was basically consistent with the hypothesis of this study. The five indicators reflecting the RUET displayed different spatial heterogeneity characteristics. In the case of the three sub-types of STS, the shrinking region along the geographical border was mainly influenced by the factors of economic aggregation, employment structure, and the income gap. Meanwhile, income gap played a dominant role in shaping shrinkage patterns in ecologically fragile regions, whereas the industrial structure and income gap were identified as key factors influencing the shrinking region adjacent to the metropolitan area. Given that the industrial structure and economic aggregation have local effects, it was suggested that these factors significantly contribute to explaining the dynamics of STS in the shrinking regions along the geographical border and adjacent to the metropolitan area (Fig 6E–6I).

The underlying reasons contributing to this phenomenon can be summarized as follows. 1. The increasing market opportunities are attributed to the strong economic ties between urban and rural areas, facilitating small towns’ access to urban markets, expanding sales prospects for local businesses, and fostering economic growth. 2. Resource sharing is facilitated by robust urban economic links in rural areas, enabling the exchange of resources such as human capital, technology, equipment, and capital. This collaboration enhances productivity in small towns while reducing production costs. 3. Strong urban-rural economic links facilitate labor mobility between cities and villages, attracting new residents and workers to small towns and mitigating population decline. 4. The substantial economic connection between urban and rural areas contributes to the diversified economic structure of small towns beyond reliance on agriculture or traditional industries. Consequently, these towns become more resilient to risks and better equipped to adapt to changing economic conditions. 5. Innovation and technology transfer are fostered through the interdependence of cities as centers of innovation and technological development with their rural counterparts. This symbiotic relationship promotes knowledge dissemination and technological advancements benefiting small towns’ competitiveness.

#### 3. Rural-urban social integration (RUSI)

Among the seven indicators in the RUSI dimension, the number of health care service staff reflecting the factor of medical service; the number of urban and rural residents enjoying minimum living allowances reflected the degree of living allowances, the per capita household consumption ratio, and the urban and rural Engel coefficient; and all these factors exhibit a negative correlation. For three sub-types of STS, the shrinking region along the geographical border was mainly affected by educational services, medical services and living allowances primarily impact the shrinking region in ecologically fragile areas, while educational services and medical services. have a major influence on the shrinking region adjacent to the metropolitan area. Educational services, medical services, and living allowances had a global effect, which indicated that all three factors had a strong effect on STS across the region. It was further observed that the population shrinkage of small towns was a unidirectional process of life-oriented movement under urbanization (Fig 6J–6P).

The underlying reasons contributing to this phenomenon can be summarized as follows. 1. Enhanced social stability and reduced contradictions and conflicts can be achieved through strong social connections between urban and rural areas, thereby making small towns a more appealing residential option for attracting and retaining residents. 2. Strong social links between urban and rural areas facilitate cultural diversity, social vitality, and effective communication, thus enhancing the attractiveness of small towns. 3. The availability of robust social support systems such as friendship networks, family ties, and comprehensive social services can significantly improve the quality of life in small towns, fostering livability. 4. Governmental support along with contributions from nonprofit organizations often reinforce the strong urban-rural ties by directing additional resources towards infrastructure development, education provision, medical services enhancement in small towns.

#### 4. Rural-urban functional identification (RUFI)

All factors, except for the level of agricultural mechanization in the RUFI dimension, exhibited a global impact with positive correlations among all indicators. Specifically, a stronger urban function was associated with higher STS, aligning with the current reality of rapid urbanization in China. In terms of functional identification within the rural system index layer, the indicator of the agricultural mechanization level demonstrated a negative correlation, with their main influencing regions being in the shrinking region along the geographical border. Here, the RUI in the context of integrated rural-urban development did not exhibit an urban bias, where cities dominated over rural areas or an equilibrium-oriented approach that disregards differentiation between urban and rural functions. Instead, it fostered interactive development while preserving individual characteristics of the urban and the rural, while preserving individual characteristics of both urban and rural domains to collectively promote regional progress. Therefore, the premise of integrated urban-rural development is based on a high degree of urbanization, industrialization, agricultural modernization and informatization, the ideal state of which should be the preservation and continuation of the significant functions for the rural region. The negative correlation between the index layer of the functional identification of the rural system and the STS also validates our hypothesis (Fig 6Q–6T).

The underlying reasons contributing to this phenomenon can be summarized as follows. 1. The development of urban centralization entails the reinforcement of urban and rural main functions, which may result in resource and opportunity concentration in major cities or urban centers, thereby attracting a larger population and increased investment while diminishing the appeal of small towns. Major cities often offer more employment opportunities, educational facilities, medical resources, etc., which allure residents from smaller towns. 2. The outflow of rural population is an outcome of strengthening urban and rural main functions as rural inhabitants are inclined to migrate towards cities in search for improved employment prospects and living conditions, which further undermines the demographic foundation of small towns. 3. There exists an uneven distribution of resources due to the bolstering of urban and rural main functions. This occasionally leads to imbalanced resource allocation with cities receiving greater government support and investment compared to insufficient development in terms of infrastructure and public services within small towns; consequently, affecting their attractiveness. 4. There is pressure associated with urbanization and modernization accompanying the enhancement of urban and rural main functions that can potentially alter people’s lifestyles and values while gradually eroding traditional culture as well as social structures within small towns.

## Discussion

China has a rich history of agrarian civilization, spanning vast territories. Small towns have long served as the economic, cultural, and social centers of rural areas, especially in the rural hinterland. China is currently experiencing rapid urbanization, characterized by a unidirectional flow from rural to urban areas. In light of this context, we put forward a hypothesis suggesting a negative relationship between the intensity of RUI and the degree of STS. Specifically, higher RUI intensity is expected to be associated with lower STS degree, and vice versa. To examine this hypothesis, we selected Henan Province in China as our case study.

In terms of their spatial pattern, a negative correlation was observed between the degree of STS and the intensity of RUI. High-value regions of STS were found to spatially coincide with low-value regions of RUI. The spatial overlay technique revealed a 59% spatial overlap between the high-value regions of STS and low-value regions of RUI as determined by the NBC method. Furthermore, our hypothesis was confirmed by the results of the MGWR analysis. 13 of the 19 evaluation indicators in the RUI system exhibited a negative correlation in the explanation of STS. It is worth noting that our study only considered shrinkage as an explanatory factor for small town development, however if growth was also considered, there would be a stronger explanatory effect on the relationship between small town development and urban-rural interaction. The extent of STS can be classified based on the surrounding geographical conditions leading to identification of three sub-type regions: the shrinking region along the geographical border, shrinking region adjacent to the metropolitan area, and the shrinking region in ecologically fragile areas.

**Fig 7:**
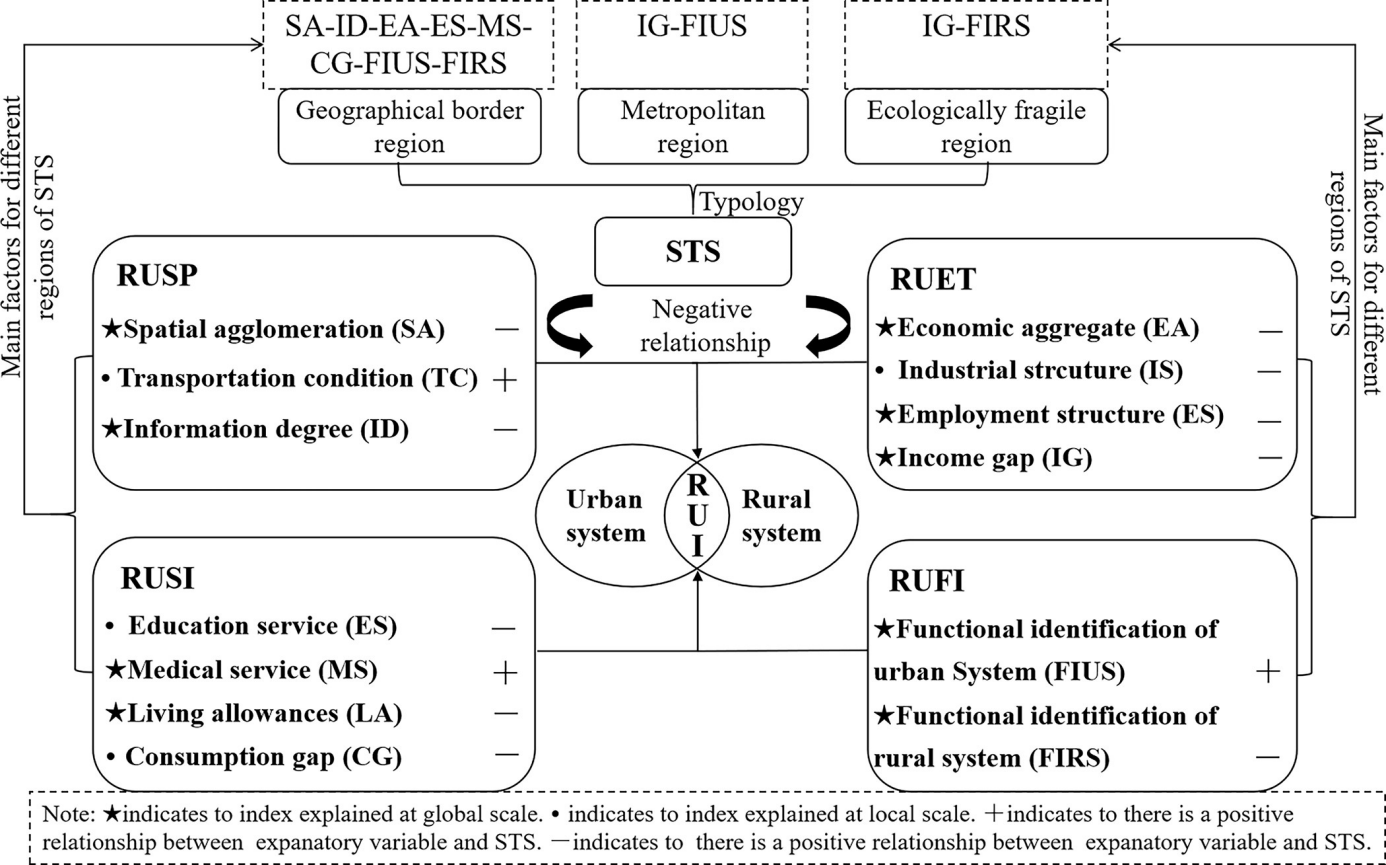
A schematic diagram showing the relationships between STS and RUI based on our results.

The factors influencing STS effectively captured the characteristics of geographical scale and spatial heterogeneity. Whether in a rural-urban territory system or an urban territory system, the STS mechanism is intricate due to the unique functions of small towns. Our work significantly contributes to this research field by expressing the spatial heterogeneity from the perspective of RUI. Among the negatively correlated indicators, there are local indicators, such as rural-urban transport convenience, income gap, employment structure, and education services. The shrinking region along the geographical border is mainly influenced by factors, such as the rural-urban income gap, employment structure, and education services. The shrinking region adjacent to the metropolitan area is predominantly influenced by the factors of rural-urban transport convenience and educational services, and the shrinking region in ecologically fragile areas was mainly influenced by rural-urban transport convenience and the income gap. To convey the these perspectives directly, we have created a schematic diagram (Fig 7).

Theoretically, STS exhibits a scale effect, which necessitates further investigation within the context of small towns maintaining their pivotal role in expanding economic networks or transforming human settlement systems. Small town shrinkage is the result of globalization, regionalization, and localization at multiple scales with varying degrees of influencing factors [42]. For example, the national context of China’s urbanization and uneven regional development has also accelerated STS in the country. The migration of rural and small-town populations towards central cities, urban agglomerations, and other regions that have witnessed rapid economic growth persists [11]. This study aims to comprehensively address the issue of STS by incorporating a systematic approach, scale, and spatial heterogeneity, thereby extending the scope beyond previous studies that solely focused on either a systematic approach and scale or a systematic approach and spatial heterogeneity. The study provided a comprehensive depiction of the macro-level phenomenon of STS, offering a holistic understanding. This approach holds an advantage as it eliminates random growth patterns and facilitates exploration into the regional characteristics inherent in STS. The disadvantage is that it does not adequately explain the cross-scale issues of STS. Subsequent studies need to be conducted to determine the factors influencing at the global scale and the impact of the spatial effect at the local scale to explain the scale shifts and scale effects of STS.

Based on the aforementioned discussion, we contend that future research could focus on the following aspects. Firstly, to enhance our comprehensive understanding of STS, forthcoming studies ought to delve deeper into cross-scale issues. This necessitates examining how factors at varying scales, ranging from local to global, interact and influence STS dynamics. Comprehending these interrelationships across different scales is pivotal for formulating effective policy interventions. Secondly, future investigations will place greater emphasis on the localized spatial effects of STS. This encompasses scrutinizing how factors such as land use, infrastructure development, and community characteristics impact the expansion or shrinkage of small towns. The local context plays a significant role in comprehending STS phenomena. Thirdly, comparative studies encompassing diverse regions can yield valuable insights into the drivers and consequences of STS. Such studies can aid in identifying best practices and lessons that can be applied in various contexts. Fourthly, research should continue to underscore policy implications by identifying effective policies capable of mitigating the adverse impacts of STS while promoting sustainable development in small towns; policymakers require evidence-based recommendations to address this issue.

## Conclusion

This study analyzed the degree, spatial association, and spatial heterogeneity of STS from 2015 to 2020 in Henan Province of China, by using the MGWR method to conduct empirical research on the factors influencing STS at the county level from the perspective of RUI. The study found that: firstly, there is a negative relationship between RUI and STS. The results showed that there was a 59% spatial overlap between the high-value regions of STS and low-value regions of RUI, which means the viewpoint was explicated in 59% of cases indicating a significant proportion of instances where the findings were elucidated. Secondly, the 59% overlap regions can be categorized into three geographical types, namely the shrinking region along the geographical border, shrinking region adjacent to the metropolitan area, and the shrinking region in ecologically fragile areas, which is also considered as the sub-types of STS. Thirdly, the characteristics of geographical scale and spatial heterogeneity were represented in the factors influencing STS from the perspective of RUI. The findings revealed spatial heterogeneity in the influencing factors across the three geographical types of STS. For instance, the STS in region adjacent to the metropolitan area is primarily driven by disparities in urban-rural income and the main function of the urban system.

The findings have contributed to a more comprehensive understanding of the formation process and mechanism of STS, thereby providing a solid foundation for formulating sustainable and holistic development policies for small towns. It is important to the type and level of support required by small towns will vary depending on their location. For instance, small towns in peripheral regions necessitate different types/levels of support compared to those located in thriving metropolitan regions. Taking the shrinking region along the geographical border in Henan Province as an example, it predominantly encompasses mountainous areas, such as Dabie, Fu Niu and Taihang mountains. These regions are characterized by high population mobility, deep poverty, and inter-provincial borders, and serve as an important ecological security barrier. Consequently, considering the diverse geographical and ecological characteristic of small towns, there should be a focus on enhancing infrastructure facilities while establishing leading industries with substantial driving effects. In summary, addressing STS-related challenges should adhere to the fundamental principles such as adapting to local conditions and promoting classification in order to effectively implement appropriate strategies based on scientific evidence.

## Supporting information

S1 File The spatial pattern of STS in Henan province. (XLSX)

S2 File The spatial pattern of the intensity of RUI. (XLSX)
